# Supplementary material for: Simultaneous Monitoring of Multi-Enzyme Activity and Concentration in Tumor Using a Triply Labeled Fluorescent In Vivo Imaging Probe
Source: Int J Mol Sci. 2020 Apr 27;21(9):3068. doi: 10.3390/ijms21093068 (PMC7246609; doi:10.3390/ijms21093068)
Supplement: Supplementary file 1 [file ijms-21-03068-s001.pdf]

Table S1. Table of synthetic peptide substrates specific for MMP2 and CatB.

Name	Peptide Sequence	Dye (N-term)	MW (g·mol^{-1})	Cleavage Site
MMP2 peptide substrate	GVPLSLYSGrrrC	AS546	2407	S–L
CatB peptide substrate	GGRRGGC	Cy5.5	1657	R–R

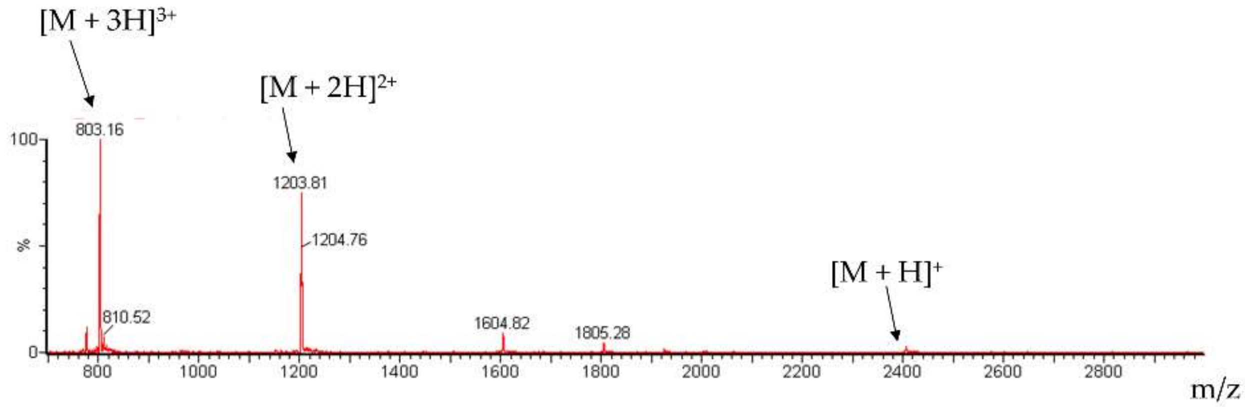

Figure S1. Mass spectrum of AF546 labeled MMP2 peptide substrate.

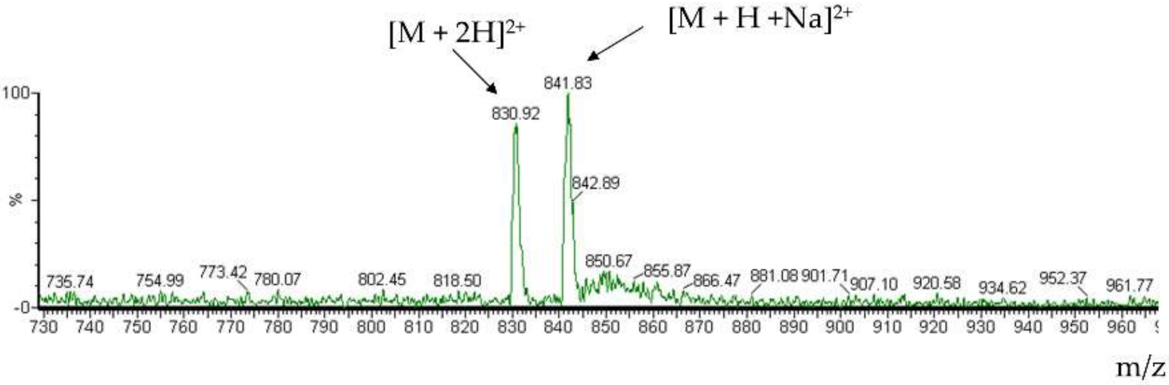

Figure S2. Mass spectrum of cy5.5 lableled CatB peptide substrate.
